# Factors Related to Masticatory Rhythm in Patients with Oral Tumors

**DOI:** 10.3390/jcm13071926

**Published:** 2024-03-26

**Authors:** Xuewei Han, Mariko Hattori, Yuka I. Sumita, Mihoko Haraguchi, Noriyuki Wakabayashi

**Affiliations:** 1 Department of Advanced Prosthodontics, Graduate School of Medical and Dental Sciences, Tokyo Medical and Dental University (TMDU), Tokyo 113-8549, Japan; han.mfp@tmd.ac.jp (X.H.); sasamfp@tmd.ac.jp (M.H.); sumita@tky.ndu.ac.jp (Y.I.S.); wakabayashi.rpro@tmd.ac.jp (N.W.); 2 Division of General Dentistry 4, The Nippon Dental University Hospital, Tokyo 102-8158, Japan

**Keywords:** masticatory rhythm, masticatory performance, maximum occlusal force, oral tumors, chewing rate, mixing ability

## Abstract

**Background:** Older adults who have undergone surgery for oral tumors are at increased risk of impaired masticatory rhythm. This study investigated the correlations between masticatory rhythm, objective masticatory performance, and subjective masticatory performance as well as factors related to masticatory rhythm. **Methods:** The participants were 44 adults (24 men, 20 women; age range 42~90 years old) who had undergone maxillectomy, mandibulectomy, or glossectomy and were rehabilitated with a maxillofacial prosthesis. The number of functional contact teeth pairs was confirmed by intraoral examination. Chewing rate, cycle duration, coefficient of variation (CV) for cycle duration (reflecting the stability of masticatory rhythm), and mixing ability were measured simultaneously using a mastication movement rhythm tracking device during gum chewing. Maximum occlusal force was measured using the dental prescale system. Patients’ perception of chewing ability was rated using a questionnaire. **Results:** The Spearman’s rank correlation test revealed that mixing ability, patient-rated masticatory scores, cycle duration, CV for cycle duration, and maximum occlusal force showed significant correlations with chewing rate. Multiple linear regression analysis identified mixing ability and the CV for cycle duration as significant predictors of masticatory rhythm. **Conclusions:** Factors associated with a faster chewing rate were higher mixing ability and masticatory scores, greater maximum occlusal force, shorter cycle duration, and smaller CV for cycle duration. Stable masticatory rhythm and mixing ability are significant predictors of chewing rate. Poor masticatory performance and unstable masticatory rhythm can result in slower chewing and thus a higher risk of inadequate dietary intake.

## 1. Introduction

Older adults with oral tumors are at greater risk of reduced food intake, malnutrition, and an unsatisfactory quality of life [1,2,3]. Patients with inadequate jaw or impaired tissues after tumor resection lose skeletal muscle mass and have fewer remaining teeth, as well as tend to chew food more slowly or swallow larger particles of food [4], all of which can make eating difficult and decrease availability of nutrients [5,6].

Masticatory rhythm, defined as masticatory frequency or chewing rate (cycles per minute or cycle duration), and the stability of masticatory frequency are important parameters in masticatory behavior and jaw movement [7,8,9,10]. Rhythmic masticatory frequency is maintained by a central pattern generator that receives input from mechanoreceptors in the periodontal ligament and masticatory muscles, facilitating mastication by coordinating with muscles of the tongue, face, and jaw to create a bolus of food for swallowing [10]. Factors reported to influence masticatory rhythm include type of occlusion, sex, temporomandibular disorders, Downs syndrome, type of food, weight of meals, and type of prosthesis [11,12]. Some researchers consider that slower chewing is beneficial in terms of making food particles smaller and recognizing items that should not be eaten despite food and energy intake being reduced, and that faster chewing is correlated with better masticatory performance and less risk of malnutrition and metabolic syndrome in individuals with natural dentition [6,8,13,14,15]. It has also been noted that improper rhythm can cause excessive strain on the temporomandibular joints, which is thought to be an etiological factor in temporomandibular disorders affecting 33% of people in Asian countries [16]. Given that masticatory frequency can be significantly affected by postoperative changes in the anatomy of the oral cavity (tongue, cheek, and mandible) [17], there is a need for studies of factors that impact masticatory rhythm in patients with oral tumors.

Masticatory function can be evaluated not only in terms of masticatory rhythm and laterality, but also masticatory performance, which itself can be evaluated objectively and subjectively [7,9,18,19,20,21]. Mixing ability is one of masticatory performances which is obtained by evaluating objective methods of the mixed color of chewed wax cube or color-changeable chewing gum and, compared with communitive ability analyzing particle size from chewed peanuts, raw carrots, or silicon rubber, is considered comparable in individuals with compromised oral status [7,9,20,21,22]. On the other hand, subjective assessment of older denture wearers’ perception of their ability to chew several types of food, determined using questionnaire consisting of 35 foods items classified into five grades based on food hardness, was found to correlate with objective masticatory performance and to be more important in maintaining oral health-related quality of life [18,23].

The timing of the parameters of jaw movement has been shown to be related to objective masticatory performance in patients with natural dentition, although conflicting results have been reported owing to lack of consistent methodology [7,8,9,17,20,21]. Moreover, no studies to date have investigated the correlation between masticatory behavior and subjective food intake ability. The purpose of this study was to determine the correlation between masticatory rhythm, mixing ability, subjective food intake ability, and contributing factors in patients with oral tumors in the hope of developing interventions and consultations for those at risk of problems with masticatory rhythm after surgery. The null hypothesis was that there would be no correlations between masticatory rhythm, mixing ability, and subjective food intake ability, and furthermore that none of the parameters investigated in this study would explain variations in masticatory rhythm.

## 2. Materials and Methods

### 2.1. Patient Eligibility

This study included 44 patients (24 men and 20 women; age range: 42~90 years old) [24], shown in Table 1, who had undergone maxillectomy, mandibulectomy, or glossectomy and subsequent rehabilitation with a maxillofacial prosthesis at Tokyo Medical and Dental University Hospital. The study was approved by the Ethics Committee of University of Tokyo Medical and Dental University (Approval No. D2021-084). Informed consent was obtained from all study participants. The main inclusion criterion was continuous wear of a maxillofacial prosthesis for at least 3 months. Patients with severe periodontal disease, a temporomandibular disorder, trismus, or disease affecting the nervous system were excluded. Information on sex, age, and number of functional contact teeth pairs were obtained by medical history-taking and intraoral examination. The number of functional contact teeth pairs was defined as erupted teeth, or replaced with crown, bridge, or implant, excluding teeth without occlusal contact, teeth that were clearly loose, and artificial teeth in dentures. The effect size was calculated using PASS 2021 software (version 21.0.3, NCSS LLC, Kaysville, UT, USA) [25].

### 2.2. Measurements

Before measurements were obtained, each participant received a detailed, face-to-face explanation of the examination procedure. Patient perception of chewing ability was rated using the food intake questionnaire by Koshino et al., which consists of 35 food items classified into five grades of masticatory difficulty based on food hardness [26]. The participants rated their ability to chew each of the 35 food items using the following scale: 0, cannot eat; 1, can eat with difficulty; and 2, can eat easily. Two additional items of “do not eat because of aversion” and “have not eaten since starting to wear dentures” were scored as 0. The scores for each of five grades were then summed to give an overall masticatory score representing perspective masticatory ability [27].

**Figure 1:**
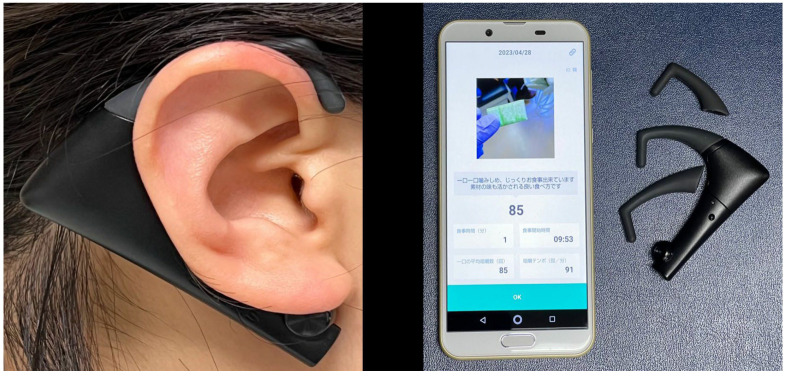
Bitescan system used to measure masticatory rhythm (Sample photo). The comments displayed on the screen after the picture emphasized, “Chewing each bite carefully allows you to enjoy your meal slowly. It’s a beneficial eating practice that also enhances the flavor of the ingredients”. Accompanying the specified chewing rate of 85 times per minute, the presented parameters included “Meal Time (minutes)”, “Meal Start Time”, “Average Number of Chews Per Bite (times)”, and “Chewing Tempo (times/min)”.

Masticatory rhythm was measured using chewing gum [22] (Xylitol^®^, 1.5 g; Lotte Co., Ltd., Tokyo, Japan) and a wearable masticatory movement tracking device (BH-BS1RR Bitescan^®^; Sharp Corp., Osaka, Japan). This device has an infrared distance sensor and accelerometer and scans the morphological change in the skin surface on the posterior side of the pinna at a mastication frequency of 20 Hz (Figure 1). The device is designed to be worn on the right side and has a variable ear-hook. A small, medium, or large ear-hook was chosen according to the size of the patient’s pinna. Before measurements were obtained, the Bitescan device was fitted on the participant’s ear to ensure that the sensor was correctly located on the back of the pinna. The Bitescan device was connected to a smartphone (SHM05, Sharp Corp.) via Bluetooth, and the data were collected using a smartphone application [28].

Each participant was instructed to sit up straight and practice chewing the gum freely for 30 s before evaluation. The participants then performed a 1-min mastication trial while wearing their prosthesis [29]. After 20 s when the chewing gum had become a soft bolus, cycle duration and the coefficient of variation (CV; standard deviation divided by the mean) thereof were obtained from the initial 10 cycles [20] as an indicator of the stability of masticatory frequency. A large deviation from the mean indicates poor masticatory performance [11,14,30]. The gum was collected immediately after chewing and compressed to a thickness of 1.5 mm in a polyethylene film between glass plates. The gum was then removed, and its color was measured through the polyethylene film using a colorimeter (CR-13; Konica-Minolta, Tokyo, Japan). Color was assessed in the CIE-L*a*b* color space. Food mixing ability (ΔE) was measured as an indicator of mixing ability; a higher score indicates greater mixing ability [20,31]. Patient satisfaction with meal intake, chewing, and overall denture status were determined using a 100-point visual analog scale [32].

**Figure 2:**
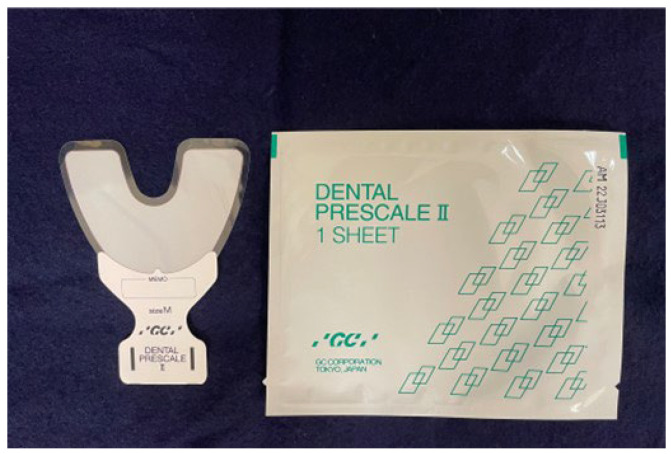
Dental Prescale device used to measure maximum occlusal force.

Maximum occlusal force (MOF) was measured on both sides using a dental prescale system that included a pressure-sensitive film (Dental Prescale 50H Type R; GC Corp., Tokyo, Japan) and an analyzer (Occluzer FDP705; GC Corp) (Figure 2). Participants held the pressure-sensitive film lightly between the maxillary and mandibular dental arches while sitting. On receiving the signal from the examiner, the participant performed maximum jaw clenching for 3 s in maximum intercuspation. Participants who used dentures were examined while wearing them. MOF in Newtons [N] of the full dental arch was calculated by scanning the pressure film using a pre-calibrated analyzer [33].

### 2.3. Statistical Analysis

Multiple linear regression was used to identify predictors of the chewing rate. Correlations between chewing rate and variables such as number of functional contact teeth pairs, MOF, mixing ability, masticatory score, and CV for cycle duration were analyzed using Spearman’s rank correlation test. The dependent variable was chewing rate, and the independent variables were functional contact teeth, MOF, mixing ability, masticatory score, and CV for cycle duration. All statistical analyses were performed using SPSS software (version 23.0; IBM Corp., Armonk, NY, USA). A sample size of 44 achieved 80% power to detect an effect size (f^2^) of 0.37 attributable to 6 independent variables using an F-Test with a significance level (α) of 0.05.

## 3. Results

### Patient Characteristics

The characteristics of participants are shown in Table 1. The results for mixing ability, masticatory score, chewing rate, cycle duration, CV for cycle duration, and MOF are shown in Table 2. The MOF were not distributed normally according to the Shapiro–Wilk test. Therefore, non-parametric tests were used for statistical analysis. The result of Spearman’s rank correlation test is shown in Table 3. Mixing ability, masticatory score, cycle duration, CV for cycle duration, and MOF showed significant correlation coefficients with chewing rate (*p* < 0.05). Mixing ability, CV for cycle duration, and chewing rate showed significant correlation coefficients with cycle duration (*p* < 0.05). Mixing ability showed a greater positive correlation with chewing rate (r = 0.476) than MOF (r = 0.411). Masticatory score, CV for cycle duration, and cycle duration showed significant correlations with chewing rate (r = 0.384, r = −0.563 and r = −0.787, respectively). Significant correlations were found among MOF, masticatory score, mixing ability, and number of functional contact teeth pairs (*p* < 0.05). Overall satisfaction with dentures showed a significant correlation with masticatory score (r = 0.460).

**Figure 3:**
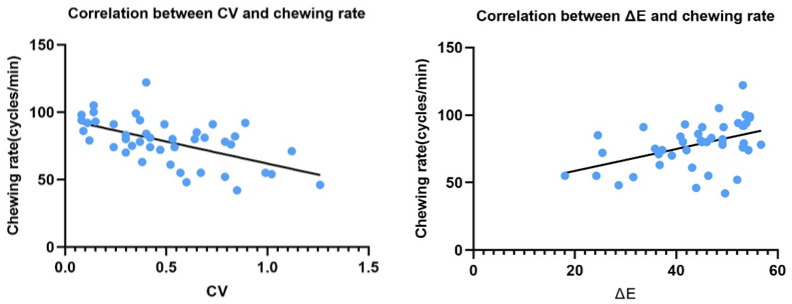
Correlation between CV, food mixing ability, and chewing rate (*n* = 44). The R^2^ value for the CV regression line was 0.329 and that for ΔE was 0.206. ΔE, food mixing ability; CV, coefficient of variation from cycle duration.

Multiple linear regression analysis identified mixing ability and CV for cycle duration to be significant predictors (*p* < 0.05) (sum R^2^ = 0.464, adjusted R^2^ = 0.375, F = 5.192, *p* < 0.001) as shown in Table 4 and Figure 3. For predictors, the variance inflation factor was less than 5 and the Durbin–Watson value was 1.761 (<2).

## 4. Discussion

This study found a significant correlation between chewing rate and masticatory performance both objectively and subjectively in patients who had undergone surgery for head and neck tumors. This finding is consistent with those of previous studies in individuals with normal dentition [7,17,21,34,35,36]. Specifically, the present study found that as masticatory frequency increased, participants showed significant improvement in masticatory performance, which is in line with previous reports of a significant positive relationship between masticatory performance and chewing rate [7,21,36], but not with others that have found an inverse correlation or no correlation between these two parameters [8,9,17,20,35,37]. The reasons for this lack of consistency are unclear. Apart from differences in design and methodology between studies (e.g., type of test food used and chewing side) [20,37], Paphangkorakit et al. suggested that a low masticatory frequency (prolonged chewing) is associated with a reduced ability to break down food into smaller particles, making it difficult to form food boluses and causing gastric distention, or is associated with an increased oral residence time causing higher levels of neuronal histamine, resulting in satiety and a further decrease in food intake [12]. Others have observed that participants with poor masticatory performance and a longer chewing cycle had a lower MOF because both occlusal force and jaw kinematics are modulated by proprioceptors and mechanoreceptors [7,38]. However, reports also indicate that participants with better masticatory performance tend to have longer cycle times (resulting in a lower chewing rate) because they slow down the rate of mandibular open–close movements, which enhances their ability to select and break down food more effectively when chewing silicon test food [17]. Furthermore, a slower chewing rate (70–90 cycles/min) is thought to allow sufficient time for electromyographic modulation, thereby improving masticatory performance [11,34]. However, these variations in chewing rate have also been attributed to differences between study populations, and the findings of these studies may not have reflected actual associations [9,17,35].

In terms of perceived ability of food intake, there was a positive correlation between food intake score and chewing rate, which is one of the parameters of masticatory behavior, whereas cycle duration, a parameter of the timing of jaw movement, showed no significant correlation. This result indicates that participants with a higher chewing rate tended to have more varied daily dietary intake. Our findings suggest that increasing masticatory frequency could be a relatively effective and simple intervention for improving both mixing ability and food intake ability in patients with maxillofacial defects, in addition to preventing malnutrition and improving their quality of life. Prosthodontists and surgeons should be aware of the potential benefits of improved chewing behavior and include it in the management of patients following treatment of oral tumors.

Among all the variables studied, the CV for cycle duration and mixing ability were the most closely correlated with masticatory frequency. CV represents the stability of jaw movement as well as neuromuscular ability in wearers of a maxillofacial prosthetic denture. Neuromuscular coordination varies from individual to individual, typically developing at a young age and often deteriorating in older age [39], resulting in diminished adaptive capacity and further influencing manual dexterity of dentures and stability of jaw movement in elderly patients. Both Uesugi et al. and Lepley et al. found a negative correlation between masticatory performance and CV for cycle duration using gummy jelly and silicon-based impression materials used as test food in participants with natural dentition, which is consistent with the results of the present study using gum in patients with oral tumors [7,8]. These findings indicate that individuals with good masticatory performance and neuromuscular coordination have a higher chewing rate and a more varied dietary intake.

Given that masticatory performance was affected by occlusal contact, occlusal force [32,40,41], and jaw movement parameters, it would be reasonable to clarify whether there is a cause-and-effect correlation of the number of functional contact teeth, MOF, and CV for cycle duration with masticatory rhythm in patients with maxillofacial defects. The correlations between MOF, number of functional contact teeth pairs, chewing rate, mixing ability, and food intake ability could be attributed to balanced occlusion, contact between cusps, adequate muscle strength, and adaptation, which result in better masticatory performance and more varied food intake [42,43]. However, a previous study found that MOF was not correlated with “eating speed” in terms of masticatory activity at mealtimes and considered that MOF is maintained by number of meals per day and appetite, not speed [41]. Nevertheless, MOF and the number of functional contact teeth pairs were not significant predictors of masticatory rhythm in our multiple linear regression model, which is consistent with previous findings [11] and indicate that these parameters are not directly correlated. Possible reasons for this finding include the fact that MOF was not generated simultaneously because chewing was performed under conditions of malocclusion, insufficient contact area, anatomical defects resulting from surgery, defect size, scarring, sensory loss, and psychological characteristics as well as potential differences in the recording techniques used [43]. Second, MOF was correlated with subjective masticatory ability and chewing rate but not with cycle duration after 20 s (Table 2). The likely reason for this finding is that the properties of the gum, when softened, are stable over 10–18 cycles after 20 s of chewing, when no additional bite force is required during constant mixing and the food bolus is stable. It has been proposed that MOF is correlated with the type of food intake and eating habits, given that different degrees of food hardness require different bite force; for example, protein and fiber require higher occlusal force and are conducive to a longer and healthier life [41,42]. The median MOF value in the present study was 234.1 N. The masseter and digastric muscles are not required to generate MOF to break down the resistance of gum, and even reduced input from the periodontal mechanoreceptors on the masticatory muscles as a consequence of inadequate dentition and loss of tissues has been considered to result in greater activity of the elevator muscles [44]. Furthermore, it is thought that there is less activation of the muscles that close the jaw, namely, the masseter and temporalis muscles, in the mixing ability test compared with the activation needed with peanuts and wax cubes used in chewing tests [21,45]. Finally, masticatory rhythm and MOF are both multifactorial variables, and while masticatory rhythm is independent of age, MOF has been found to decrease with aging [40,41]. Multiple test foods and larger sample sizes with different maxillofacial disorders will be needed in further studies. In terms of the number of functional contact teeth pairs, all our participants required rehabilitation with a maxillofacial prosthesis. Some studies have suggested that intervention with a prosthesis could restore masticatory rhythm to some extent by establishing an adequate dental arch and stabilizing jaw movement [46,47]. However, restoration of MOF is limited under denture-wearing conditions [23]. Our patients who adapted well to dentures by manipulating the tongue and cheek to retain the denture during use were very satisfied with their dentures, especially in terms of meal intake and chewing (Table 1). Therefore, the number of functional contact teeth pairs might not have reflected the actual association with masticatory rhythm in the present study.

This study had some limitations. Masticatory rhythm and MOF are affected by patient willingness to complete the measurement activities as instructed and the recording techniques used [11,43]. Therefore, measurements should have been repeated to reduce variation in masticatory rhythm. Given the various types of prosthesis retention and surgical reconstruction that can be considered, such as maxillary perforations, flap reconstruction, or metal plate reinforcement in the mandible, it is important to note that flap-supported dentures might interfere with denture stability and potentially affect the masticatory rhythm [11,46,47]. Future studies may, therefore, categorize prostheses based on these considerations. Chewing patterns were variable because some patients were wearing both upper and lower dentures, characterized by small vertical amplitude and lateral excursion, presented with a chopping chewing pattern, whereas patients who were wearing a single-jaw denture were likely to exhibit a grinding chewing pattern or a combination of chopping and grinding [47]. Adjuvant neck dissection has been suggested to affect mandibular kinematics [43], and its unwanted effects, such as fibrosis, and radiotherapy-induced xerostomia should be investigated in a larger sample size in future.

In terms of clinical implications, our findings provide a better understanding of the role of masticatory rhythm in objective masticatory performance and perceived chewing ability in older patients with oral tumors, allowing more detailed consultations for individualized prosthetic and nutritional interventions. They also highlight the need for clinical measurement of masticatory rhythm and routine follow-up.

## 5. Conclusions

The factors associated with a faster chewing rate were higher mixing ability and masticatory scores, higher MOF, shorter cycle time, and lower CV for cycle duration. Stability of masticatory rhythm and mixing ability are significant predictors of chewing rate. Poor masticatory performance and unstable masticatory rhythm can lead to a slower chewing rate and thus carry a higher risk of inadequate dietary intake.

## Figures and Tables

**Table 1:** Characteristics of the participants (*n* = 44).

Characteristics	Frequency/Years
Age range (42~90 years old)	44
Middle-aged adults (40~59 years old)	6
Old-aged adults (60~years old ≤)	38
Surgery procedure	
Maxillectomy	24
Mandibulectomy	19
Glossectomy	5
Tongue cancer without surgery	2
Diagnosis	
Benign	7
Malignant	37
Rehabilitation period after surgery	2 years~46 years 3 months

**Table 2:** Median and range for variables (*n* = 44).

Variables	Median	Range
Chewing rate (cycles/min)	80.00	42.00–122.00
Cycle duration (s)	0.67	0.41–2.50
CV for cycle duration	0.44	0.00–1.00
Maximum occlusal force (N)	234.10	8.50–1010.80
Functional contact teeth pairs (*n*)	6.00	0–14.00
Satisfaction with mixing ability	45.55	18.00–56.70
Masticatory scores	95.33	34.63–100.04
Meal intake	80.00	30.00–100.00
Satisfaction with chewing	80.00	30.00–100.00
Overall satisfaction with denture	90.00	20.00–100.00

CV, coefficient of variation.

**Table 3:** Spearman’s correlation coefficients for variables (*n* = 44).

	Cycles/min	Cycle Duration	CV	Masticatory Score	ΔE	Functional Contact Teeth Pairs (*n*)	Overall Satisfaction with Denture
Cycle duration	−0.787 **						
CV	−0.563 **	0.503 **					
Masticatory score	0.384 *	−0.268	−0.403 **				
ΔE	0.476 **	−0.321 *	−0.253	0.326 *			
Functional contact teeth pairs (*n*)	0.284	−0.051	−0.161	0.413 **	0.428 **		
Overall satisfaction with denture	0.007	0.009	0.025	0.460 **	0.245	0.210	
MOF	0.411 **	−0.144	−0.181	0.518 **	0.412 **	0.445 **	0.236

** Correlation was significant at the 0.01 level (2-tailed). * Correlation was significant at the 0.05 level (2-tailed). A sample size of 44 achieved 80% power to detect an effect size (f^2^) of 0.37 attributable to 6 independent variables using an F-test with a significance level (α) of 0.05. ΔE, food mixing ability; CV, coefficient of variation for cycle duration; MOF, maximum occlusal force.

**Table 4:** Multiple linear regression model for chewing rate (*n* = 44).

Dependent Variable	Independent Variable	B	β	*p*-Value
Chewing rate	Intercept	65.837	−	0.00
	CV	−23.423	−0.411	0.006 **
	ΔE	0.579	0.325	0.035 *
	Masticatory score	0.125	0.133	0.449
	Functional contact teeth pairs (*n*)	0.398	0.088	0.549
	MOF	−0.001	−0.014	0.926
	Overall satisfaction with denture	−0.156	−0.198	0.188

A sample size of 44 achieved 80% power to detect an effect size (f^2^) of 0.37 attributable to 6 independent variables using an F-test with a significance level (α) of 0.05. R^2^ = 0.464, adjusted R^2^ = 0.375, F = 5.192, *p* < 0.001. B, partial regression coefficient. β, standardized partial regression coefficient. ΔE, food mixing ability; CV, coefficient of variation for cycle duration; MOF, maximum occlusal force. * *p* < 0.05, ** *p* < 0.01.

## Data Availability

Data cannot be shared publicly because the data are owned and saved by Tokyo Medical and Dental University. Data are available from the Tokyo Medical and Dental University Ethics Committee for researchers who meet the criteria for access to confidential data: contact address, pararotti.mfp@tmd.ac.jp.
